# Etiology and severity of diarrheal diseases in infants at the semiarid region of Brazil: A case-control study

**DOI:** 10.1371/journal.pntd.0007154

**Published:** 2019-02-08

**Authors:** Aldo A. M. Lima, Domingos B. Oliveira, Josiane S. Quetz, Alexandre Havt, Mara M. G. Prata, Ila F. N. Lima, Alberto M. Soares, José Q. Filho, Noélia L. Lima, Pedro H. Q. S. Medeiros, Ana K. S. Santos, Herlice N. Veras, Rafhaella N. D. G. Gondim, Rafaela C. Pankov, Mariana D. Bona, Francisco A. P. Rodrigues, Renato A. Moreira, Ana C. O. M. Moreira, Marcelo Bertolini, Luciana R. Bertolini, Vicente J. F. Freitas, Eric R. Houpt, Richard L. Guerrant

**Affiliations:** 1 INCT-Biomedicine and Department of Physiology and Pharmacology, Faculty of Medicine, Federal University of Ceará, Fortaleza, CE, Brazil; 2 Faculty of Pharmacy, University of Fortaleza, Fortaleza, CE, Brazil; 3 Faculty of Veterinary Medicine, State University of Ceará, Fortaleza, CE, Brazil; 4 Center for Global Health and Division of Infectious Diseases and International Health, University of Virginia, Charlottesville, VA, United States of America; Johns Hopkins University Bloomberg School of Public Health, UNITED STATES

## Abstract

**Background:**

Diarrheal diseases are an important cause of morbidity and mortality among children in developing countries. We aimed to study the etiology and severity of diarrhea in children living in the low-income semiarid region of Brazil.

**Methodology:**

This is a cross-sectional, age-matched case-control study of diarrhea in children aged 2–36 months from six cities in Brazil’s semiarid region. Clinical, epidemiological, and anthropometric data were matched with fecal samples collected for the identification of enteropathogens.

**Results:**

We enrolled 1,200 children, 596 cases and 604 controls. By univariate analysis, eight enteropathogens were associated with diarrhea: Norovirus GII (OR 5.08, 95% CI 2.10, 12.30), Adenovirus (OR 3.79, 95% CI 1.41, 10.23), typical enteropathogenic *Escherichia coli* (tEPEC), (OR 3.28, 95% CI 1.39, 7.73), enterotoxigenic *E*. *coli* (ETEC LT and ST producing toxins), (OR 2.58, 95% CI 0.99, 6.69), rotavirus (OR 1.91, 95% CI 1.20, 3.02), shiga toxin-producing *E*. *coli* (*STEC*; OR 1.77, 95% CI 1.16, 2.69), enteroaggregative *E*. *coli* (EAEC), (OR 1.45, 95% CI 1.16, 1.83) and *Giardia* spp. (OR 1.39, 95% CI 1.05, 1.84). By logistic regression of all enteropathogens, the best predictors of diarrhea were norovirus, adenovirus, rotavirus, STEC, *Giardia* spp. and EAEC. A high diarrhea severity score was associated with EAEC.

**Conclusions:**

Six enteropathogens: Norovirus, Adenovirus, Rotavirus, STEC, *Giardia* spp., and EAEC were associated with diarrhea in children from Brazil’s semiarid region. EAEC was associated with increased diarrhea severity.

## Author summary

Most childhood diarrheal diseases studies focus on children from health centers or emergency hospitals that contribute to a lack of understanding of the etiology of community diarrhea. We aimed to investigate the etiology and severity of diarrheal diseases in children living in Brazil’s low-income, semiarid region communities. We used a case-control study of diarrhea (1,200 children, 596 cases and 604 controls) and showed that six enteropathogens (norovirus, adenovirus, rotavirus, STEC, Giardia and EAEC) are associated with diarrhea in these communities. Furthermore, enteroaggregative *E*. *coli* (EAEC) was associated with a high diarrhea clinical severity score. These findings help further the understanding of diarrheal disease etiology of previously unevaluated children from Brazil’s low-income semiarid region. This study contributes new information that expands our knowledge of diarrheal etiology in non-hospitalized children from developing countries. Moreover, we believe this work will support further research on specific enteropathogens, with special attention to the role of EAEC in severe cases of diarrhea and guide public health policies and physicians in the management of diarrheal diseases in Brazil’s low-income semiarid region.

## Introduction

Diarrheal diseases remain a prominent cause of morbidity and mortality in developing countries as the second most common cause of death in children under five years old [1,2]. Studies of diarrheal illness among children in Brazil and other developing countries has focused on health centers or emergency hospitals that primarily treat patients with moderate-to-severe diarrhea and have the ability to identify enteropathogens [3–5]. This approach captures only a small subset of Brazil’s diarrheal diseases burden and limits accurate understanding about pathogen prevalence in the poorest semiarid region of Brazil.

The high prevalence of diarrheal disease in developing areas is significant because it inhibits normal growth, impairs cognitive function, and disrupts physical and educational development in children [6–14]. Therefore, enteropathogen specific studies of diarrheal diseases etiologies and clinical severity in Brazil’s semiarid region will contribute to public health preventions and interventions against diarrheal diseases.

This study is a cross-sectional, multisite work that evaluated childhood diarrheal etiology among six cities in Brazil’s semiarid region. We aimed to investigate etiology, severity of diarrheal episodes, and environmental factors associated with diarrheal diseases in children 2–36 months old.

## Materials and methods

### Study design, setting and enrollment criteria

A case-control study was conducted in six cities with a population greater than fifty-thousand that were randomly selected from the five states of Brazil’s semiarid region: Cajazeiras (Paraíba), Crato (Ceará), Ouricuri (Pernambuco), Patos (Paraíba), Picos (Piauí) and Sousa (Paraíba). During the active surveillance period from November 2009 to February 2012, fecal samples were collected from children aged 2–36 months who reside in urban communities near primary health care units. Cases were defined as children with diarrhea (three or more liquid stools in the last 24 hours). A standardized questionnaire was completed during the enrollment interview to collect the following detailed health information: demographic, environmental, socio-economic status, breastfeeding practices, other clinical conditions, vaccination history, frequency of diarrhea episodes and anthropometric measurements.

Diarrhea cases were finding via active surveillance by field workers walk door-door in the vicinity of the primary health care units and investigate the households until the sample size was reached. Diarrhea cases were defined as a child aged 2–36 months with a history of three or more liquid stools in the last 24 hours prior to the arrival of fieldworkers who were responsible to collect the stool samples. Inclusion criteria (diarrhea cases) were: 1) had three or more liquid stools in the last 24-hours; 2) had no chronic illness or hospitalization within 12-hours of study enrollment; and 3) written consent provided by parents or legal guardians. Inclusion criteria for no-diarrhea, controls, were: 1) did not present with diarrhea in the past two weeks; and 2) written consent provided by parents or legal guardians

### Semiarid region in Brazil

**Fig 1:**
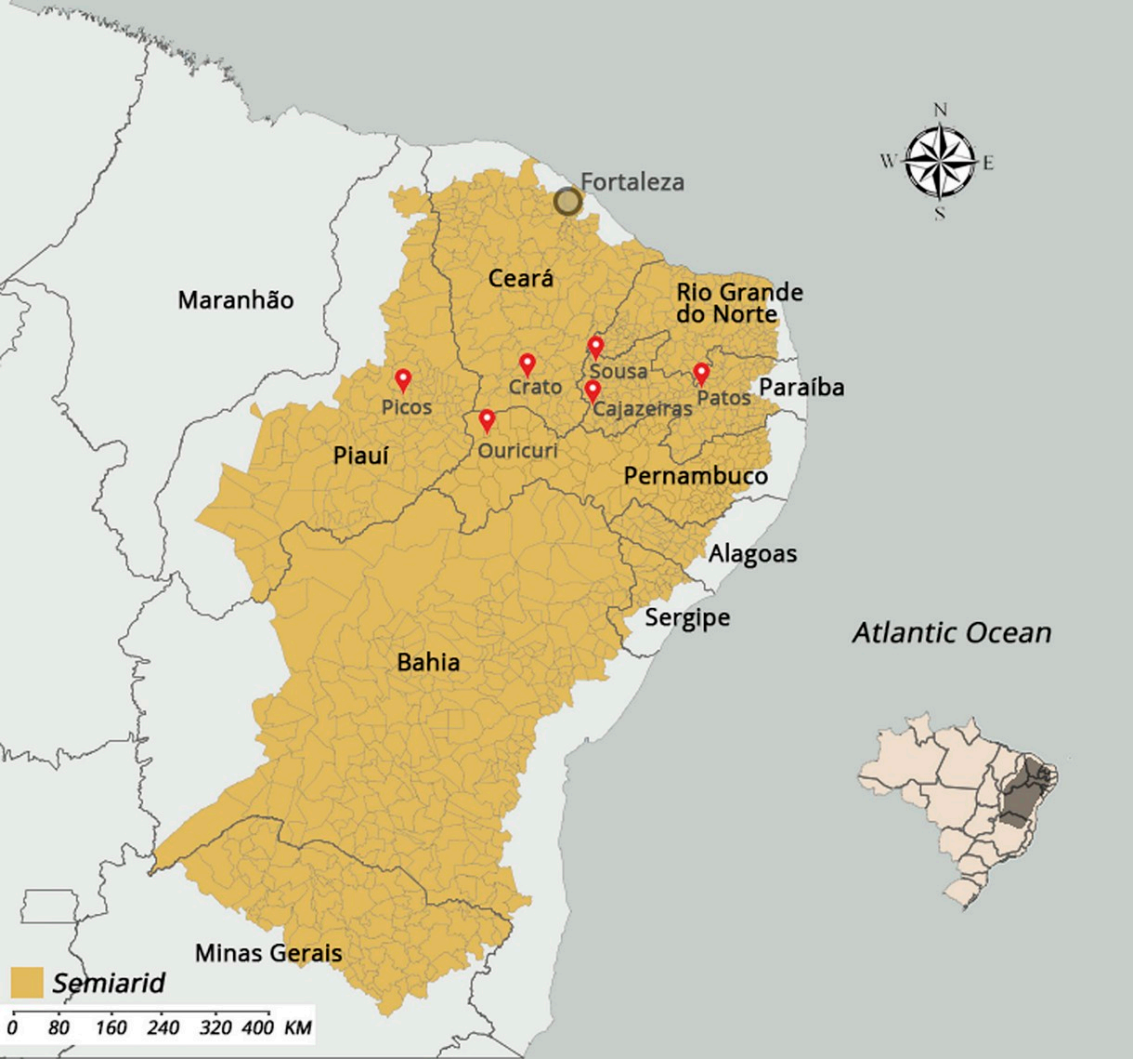
Location, participant enrollment and selection. The semiarid region includes the states of Ceará, Piauí, Rio Grande do Norte, Paraíba, Pernambuco, Alagoas, Sergipe, and Bahia of the Northeastern macro-region, but does not include Maranhão and the area north of Minas Gerais (Fig 1). The semiarid region covers 969,589.4 km^2^ and has a population of 23.5 million. The estimated population of children under five years old is 2.3 million. The average annual rainfall is less than 800 mm and the aridity index can reach 0.5, which represents the water balance between precipitation and potential evapotranspiration. Drought risk in the semiarid region is greater than 60% [15].

The semiarid region includes the states of Ceará, Piauí, Rio Grande do Norte, Paraíba, Pernambuco, Alagoas, Sergipe, and Bahia of the Northeastern macro-region, but does not include Maranhão and the area north of Minas Gerais (Fig 1). The semiarid region covers 969,589.4 km^2^ and has a population of 23.5 million. The estimated population of children under five years old is 2.3 million. The average annual rainfall is less than 800 mm and the aridity index can reach 0.5, which represents the water balance between precipitation and potential evapotranspiration. Drought risk in the semiarid region is greater than 60% [15].

### Demographic, epidemiology, environmental and socio-economic status

Case report forms were designed to collect information during child enrollment and capture demographic, birthdate, sex, anthropometric measurements such as current weight, length and head circumference, child care practices such as breastfeeding, and the characteristics of the mother/caregiver. Environmental and socio-economic status data capture included: household exterior material, number of rooms, number of people sleeping in the household, number of children less than five years old, source of drinking water, toilet facilities, number/type of animals living in their household, and the average monthly income for the entire household.

### Clinical and vaccination information collected

Clinical data were collected at the time of enrollment and diarrhea episodes were identified by the fieldworkers. They were defined as a maternal report of three or more liquid stools in a 24-hour period. Discrete episodes had at least two intervening days without diarrhea. A diarrhea severity score was adapted for every episode using elements derived from the Malnutrition-Enteric Disease MAL-ED scores [16]. Dehydration was defined as moderate or severe based on the World Health Organization manual of the treatment of diarrhea [17]. Dysentery was defined as the presence of visible blood in the stool as reported by the child´s mother/caregiver. Diarrhea associated with fever was defined as diarrhea and the mother recording a temperature greater than 37.5°C. Vomiting associated diarrhea was defined as vomiting at any point during an episode of diarrhea. Vaccine administration data was captured for the following: rotavirus (Rotarix G1P[8] GlaxoSmithKline, Wavre, Belgium); BCG: Bacillus Calmette-Guérin; MMR: Measles, Mumps and Rubeola; Hepatitis B; Hib: *Haemophilus influenzae* type b; DPT: Diphtheria, Pertussis, Tetanus; and OPV: Oral Polio Vaccine. In addition, antibiotic and other medications given to the child during diarrhea episodes were recorded.

### Anthropometry measurements

The study protocol used a standard recumbent length measuring board (Schorr Productions, Olney, MD) to measure the length of children to the nearest 0.1 cm. Digital scales were also used to measure weight to the nearest 100 g. The weight-for-age (WAZ), length-for-age (LAZ), and weight-for-length (WLZ) z-scores were calculated using the World Health Organization Multi-Country Growth Reference Study [18]. This study used a Seca 212 infant head circumference tape (Seca Deutschland, Hamburg, Germany) made of non-stretch Teflon synthetic material and a range of 5–59 cm marked in 0.1 cm increments

### Molecular diagnostics for enteropathogens and inflammatory biomarkers

Stool specimens were collected and stored at -80°C until used. DNA and RNA extraction were performed using the QIAamp DNA Stool Mini Kit (Qiagen, USA) and QIAamp Viral RNA extraction kit (QIAGEN, USA), respectively. Nucleic acid was amplified with sequence-specific primer-probe sets (S1 Table). Either forward or reverse primers were biotinylated on the 5’-end and probes were amine-modified at the 5′-end comprised of 12-carbon spacers to enable coupling to the carboxylated fluorescent microspheres. Following Multiplex polymerase chain reaction (PCR) reactions and membrane hybridization procedures, samples were analyzed by the Bio-Plex 200 System (Bio-Rad, CA, USA) [19,20]. The results were reported as microsphere specific media fluorescent intensity (MFI) and corrected for background bead fluorescence. Corrected MFI were calculated as follows: cMFI = (MFIanalyte−MFI_negative control_) / MFI_negative control_. Positive samples had cMFI values greater than three. Positive (DNA template from reference organisms) and negative controls (nuclease-free water) were included in every run. We used four distinct Multiplex PCR panels (bacteria 1 and 2, protozoa and virus) to identify 17-different enteropathogens. All PCR conditions and references are described in S1 Table. Bead coupling and hybridization procedures followed descriptions published elsewhere [19,20]. Myeloperoxidase (MPO) biomarkers were measured in stool samples to access gut inflammation using a kit from Immunodiagnostic (Bensheim, Germany).

### Ethics statement

The study protocol and consent form were approved by the local institutional review board (IRB) at all cities sites and by collaborating IRBs, and approved by Brazil’s National Commission on Ethics in Research and the Research Ethics Committee of the Federal University of Ceará (Craft No. 5502006, Protocol No. 23805). Written informed consent was obtained from the parent or guardian of every child.

### Sample size and statistical analysis

The estimated sample size of infantile diarrhea etiology in the Brazilian semiarid region was 980 to 1,400 children. The sample size of 278 cases and 278 controls provided a statistical power of 80% and a statistical significance of P <0.05 for pathogen isolation in at least 6% of cases and 1.5% of controls. We estimated a 10% loss of subjects from the study thereby requiring we have 306 cases and 306 controls for a total of 612 subjects for the study.

The collected data were entered into Excel spreadsheets v.4.0 (Microsoft Corp., Seattle, WA) by two independent data entry persons and then compared to ensure accuracy. Statistical analysis was performed using SPSS (IBM Corp. Released 2013. IBM SPSS Statistics for Windows, Version 20.0. Armonk, NY: IBM Corp.) and used for all analyses. All study subject samples and data were analyzed anonymously. The Shapiro-Wilk test was used to evaluate the normality of the quantitative variable data, and the Levene test was used to evaluate the equality of the variances. The Student's t test was used for normally distributed variables; the Mann-Whitney test (two groups) and Kruskal Wallis test (three or more groups) were used for variables whose distribution was not normal. Qualitative variables were analyzed using the chi-square test or Fisher's test. GraphPad Prism software, version 3.0 for Windows (San Diego, CA, USA), was used for complementary statistical analysis, table formatting and figures. Multivariate logistic regression models were used to access risk factors and identify the enteropathogens most associated with diarrhea episodes. Factors of risk or protection included child anthropometrics, child care, mother/caregiver characteristics, environmental and socio-economic parameters. We also used multivariate logistic regression models to access etiologic association with the following outcomes: diarrhea severity, signs and symptoms, and episode duration. In these models, values for β coefficients (SE) were presented to show the positive or negative relationship between the variable and the outcome. Odds ratios (OR) with 95% confidence intervals (95% CI) were utilized to assess the risk between a variable and its outcome. A significance level of <0.05 was used for all statistical analyses.

## Results

A total of 1,600 children were screened, 400 were ineligible and 1,200 children were enrolled (596 cases and 604 controls). All 1,200 children provided stool samples and their data are detailed in Table 1.

**Table 1:** Selected baseline characteristics of the diarrhea cases and controls children included in the univariate analysis.

Variables	Total	Diarrhea	Controls	*P* values
	N = 1200	N = 596	N = 604	
**Child anthropometrics**				
Age (months; mean ± sem)	18.1 ± 0.28	16.8 ± 0.40	19.4 ± 0.38	<0.001
Male (n/Total; %)	624 (52)	312 (52)	312 (52)	0.817
Current weight of the child (mean ± sem)	10.8 ± 0.08	10.5 ± 0.11	11.2 ± 0.11	<0.001
Current length of the child (mean ± sem)	79.9 ± 0.30	77.2 ± 0.45	80.7 ± 0.39	<0.001
Current head circumference (mean ± sem)	46.8 ± 0.09	46.4 ± 0.14	47.2 ± 0.12	<0.001
**Child care**				
Is your child still breastfeeding (mixed or exclusive) him/her? (n ≥2 days / Total; %)	568 (47)	333 (56)	235 (39)	<0.001
**Characteristics of the mother/caregiver**				
(Mother) How many years of schooling have you completed? (N = incomplete 8 years of school/Total; %)	889 (74)	452 (76)	437 (73)	0.260
Age of the mother at child enrollment	26.2 ± 0.18	25.6 ± 0.26	26.7 ± 0.26	0.002
Age of your first pregnancy? (mean ± sem)	20.2 ± 0.14	19.88 ± 0.20	20.5 ± 0.20	0.012
**Socio-economic status**				
Main material of the household exterior? (N = cement or concrete/Total; %)	1157 (97)	570 (96)	587 (98)	0.082
How many rooms are there in your household? (mean ± sem)	4.8 ± 0.05	4.68 ± 0.06	4.99 ± 0.07	0.007
How many people usually sleep in this household? (mean ± sem)	4.5 ± 0.05	4.48 ± 0.07	4.52 ± 0.07	0.578
How many children less than 5 years old sleep in this household? (mean ± sem)	1.31 ± 0.016	1.34 ± 0.02	1.29 ± 0.02	0.132
What is the main source of drinking water for members of your household? (N = piped into dwelling or to yard/plot or public tap/stand pipe/Total; %)	1168 (98)	583 (98)	585 (97)	0580
What you do before drinking the water? (N = filter/boiled/or chlorination/Total; %)	870 (73)	424 (71)	446 (74)	0.302
What kind of toilet facility do members of your household usually used? (N = flush to piped server system or septic tank/Total; %)	1144 (95)	567 (95)	577 (96)	0.785
Do you have animal in your household? (N = yes/Total; %)	416 (35)	196 (33)	220 (36)	0.203
What is the average monthly income for the entire household? (mean ± sem)	2.83 ± 0.03	2.80 ± 0.04	2.85 ± 0.05	0.642

The Student *t* test was used for normally distributed variables and Mann-Whitney test for variables whose distribution was not normal and Chi-square analysis was used for contingency. SEM = standard error of mean.

**Fig 2:**
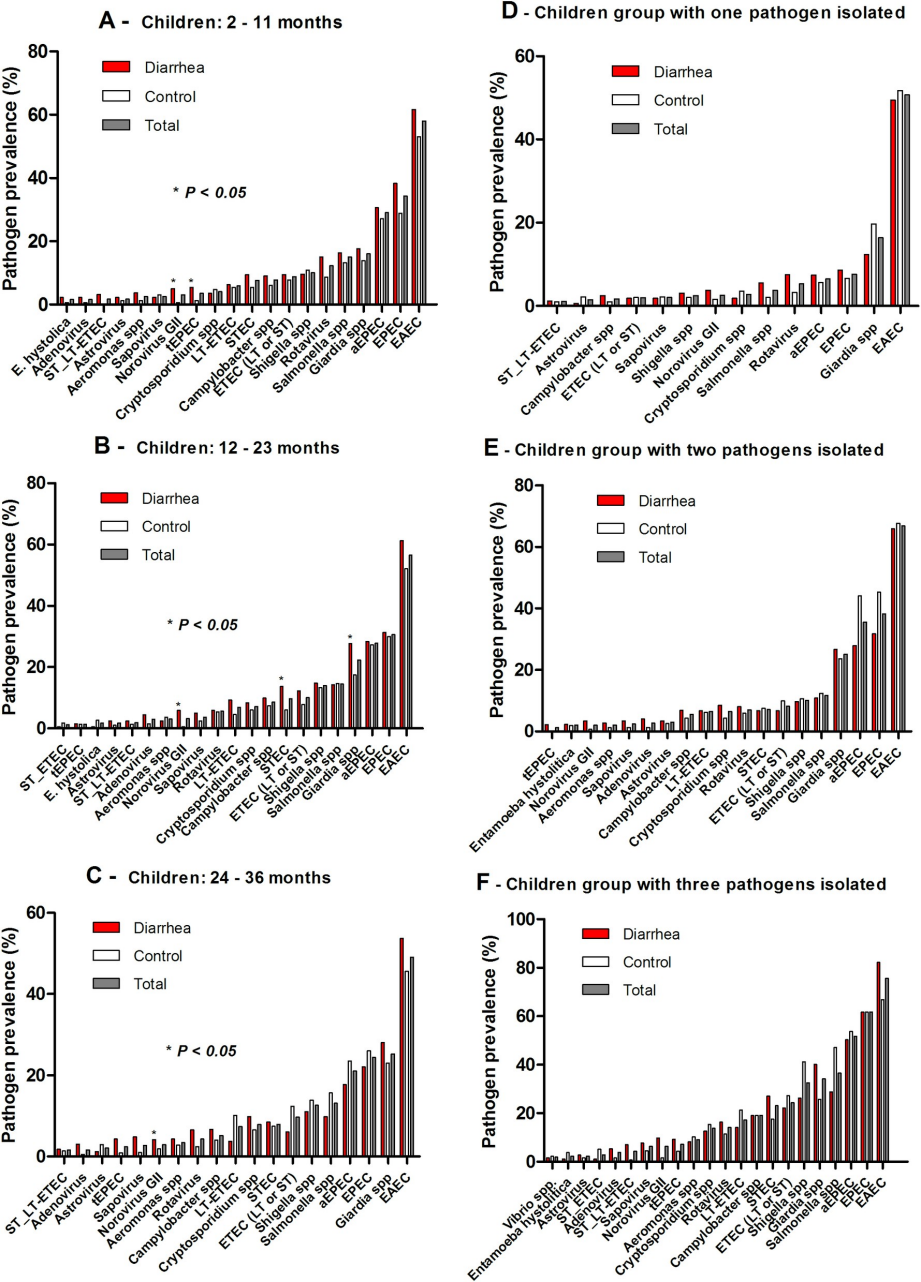
**Enteropathogens detected in diarrheal and non-diarrheal stools from children: A) 2–11 months, B) 12–23 months, and C) 24–36 months; pathogen prevalence by grouping: D) children with one, E) two, and F) three or more pathogens from the semiarid region in Brazil**. EAEC = enteroaggregative *E*. *coli*; STEC = shiga toxin-producing *E*. *coli*; tEPEC = typical enteropathogenic *E*. *coli*; ST or LT ETEC = heat-stable or heat-labile producing enterotoxigenic *E*. *coli*.

The overall prevalence of enteropathogens in diarrhea cases and controls are summarized in Table 2. Pathogen prevalence by first, second and third year of life are shown in Fig 2. Norovirus GII (OR = 8.514; 95% CI: 1.088–66.632) and typical enteropathogenic *Escherichia coli* (tEPEC), (OR = 4.686; 95% CI: 1.034–21.228) were significantly associated with the diarrhea case group compared to control children in the first year of life (Fig 2A). In the second year of life norovirus GII (OR = 12.758; 95% CI: 1.643–99.061), shiga toxin-producing *Escherichia coli* (STEC), (OR = 2.497; 95% CI: 1.255–4.967) and *Giardia* spp. (OR = 1.807; 95% CI: 1.133–2.880) were significantly associated with diarrheal episodes (Fig 2B). In the third year we only identified s*apovirus* (OR = 5.215; 95% CI: 1.092–24.900), which had a significant association with the diarrhea case group when compared to the controls (Fig 2C).

**Table 2:** Prevalence of enteropathogens in diarrhea cases and controls children from the semiarid region in Brazil.

Enteropathogens	Total prevalence, N / Total (%)	Diarrhea, N / Total (%)	Controls, N / Total (%)	*P* values	OR	95% CI
**Virus**						
Rotavirus	86 /1161 (7.4)	56 / 588 (9.5)	30 / 573 (5.2)	0.007	1.905	1.204–3.016
Norovirus *GII*	36 / 1161 (3.1)	30 / 588 (5.1)	6 / 573 (1.0)	<0.001	5.081	2.098–12.301
Sapovirus	35 / 1161 (3.0)	23 / 588 (3.9)	12 / 573 (2.1)	0.86	1.903	0.938–3.862
Adenovirus	24 / 1161 (2.1)	19 / 588 (3.2)	5 / 573 (0.9)	0.006	3.793	1.407–10.229
Astrovirus	22 / 1161 (1.9)	12 / 588 (2.0)	10 / 573 (1.7)	0.831	1.173	0.503–2.737
**Bacteria**						
EAEC (*aatA* or *aaiC)*	650 / 1191 (54.6)	350 / 591 (59.1)	300 / 600 (50.0)	0.002	1.452	1.155–1.826
EPEC (*bfA* or *eaeA*)	355 /1191 (29.8)	185 / 591 (31.3)	170 / 600 (28.3)	0.282	1.153	0.899–1.478
*aEPEC* (*eaeA*)	310 / 1191 (26.0)	155 / 591 (26.2)	155 / 600 (25.8)	0.895	1.021	0.788–1.322
*tEPEC* (*bfA*)	29 / 1191 (2.4)	22 / 591 (3.7)	7 / 600 (1.2)	0.004	3.275	1.388–7.727
*Salmonella* spp. (*invA*)	169 / 1187 (14.2)	81 / 587 (13.8)	88 / 600 (14.7)	0.679	0.931	0.672–1.290
*Shigella* spp. (*ipaH*)	147 / 1187 (12.4)	70 / 587 (11.9)	77 / 600 (12.8)	0.660	0.920	0.651–1.299
ETEC (LT or ST) (*eltB* or *estA)*	113 / 1191 (9.5)	56 / 591 (9.5)	57 / 600 (9.5)	1.00	0.997	0.677–1.469
*LT-ETEC* (*eltB*)	80 / 1191 (6.7)	39 / 591 (6.6)	41 / 600 (6.8)	0.908	0.963	0.612–1.517
*ST-ETEC* (*estA*)	12 / 1191 (1.0)	2 / 591 (0.3)	10 / 600 (1.7)	0.038	0.200	0.044–0.918
*ST_LT-ETEC* (*estA* and *eltB)*	21 / 1191 (1.8)	15 / 591 (2.5)	6 / 600 (1.0)	0.049	2.578	0.993–6.691
STEC (*stx1* or *stx2)*	101 / 1191 (8.5)	63 / 591 (10.7)	38 / 600 (6.3)	0.009	1.765	1.160–2.685
*Campylobacter* spp. (*cadF*)	86 / 1187 (7.2)	51 / 587 (8.7)	35 / 600 (5.8)	0.073	1.536	0.983–2.400
*Aeromonas* spp. (*aerA*)	36 / 1187 (3.0)	20 / 587 (3.4)	16 / 600 (2.7)	0.501	1.287	0.660–2.510
*Vibrio* spp. (*toxR*)	7 / 1187 (0.6)	4 / 587 (0.7)	3 / 600 (0.5)	0.723	1.365	0.304–6.127
**Protozoa**						
*Giardia* spp.	252 / 1187 (21.2)	141 / 587 (24.0)	111 / 600 (18.5)	0.023	1.393	1.053–1.843
*Cryptosporidium* spp.	76 / 1187 (6.4)	41 / 587 (7.0)	35 / 600 (5.8)	0.477	1.212	0.761–1.932
*Entamoeba histolytica*	15 / 1187 (1.3)	6 / 587 (1.0)	9 / 600 (1.5)	0.605	0.678	0.240–1.917

All primers, polymerase chain reaction conditions and references are described in **S1 Table**.

**Fig 3:**
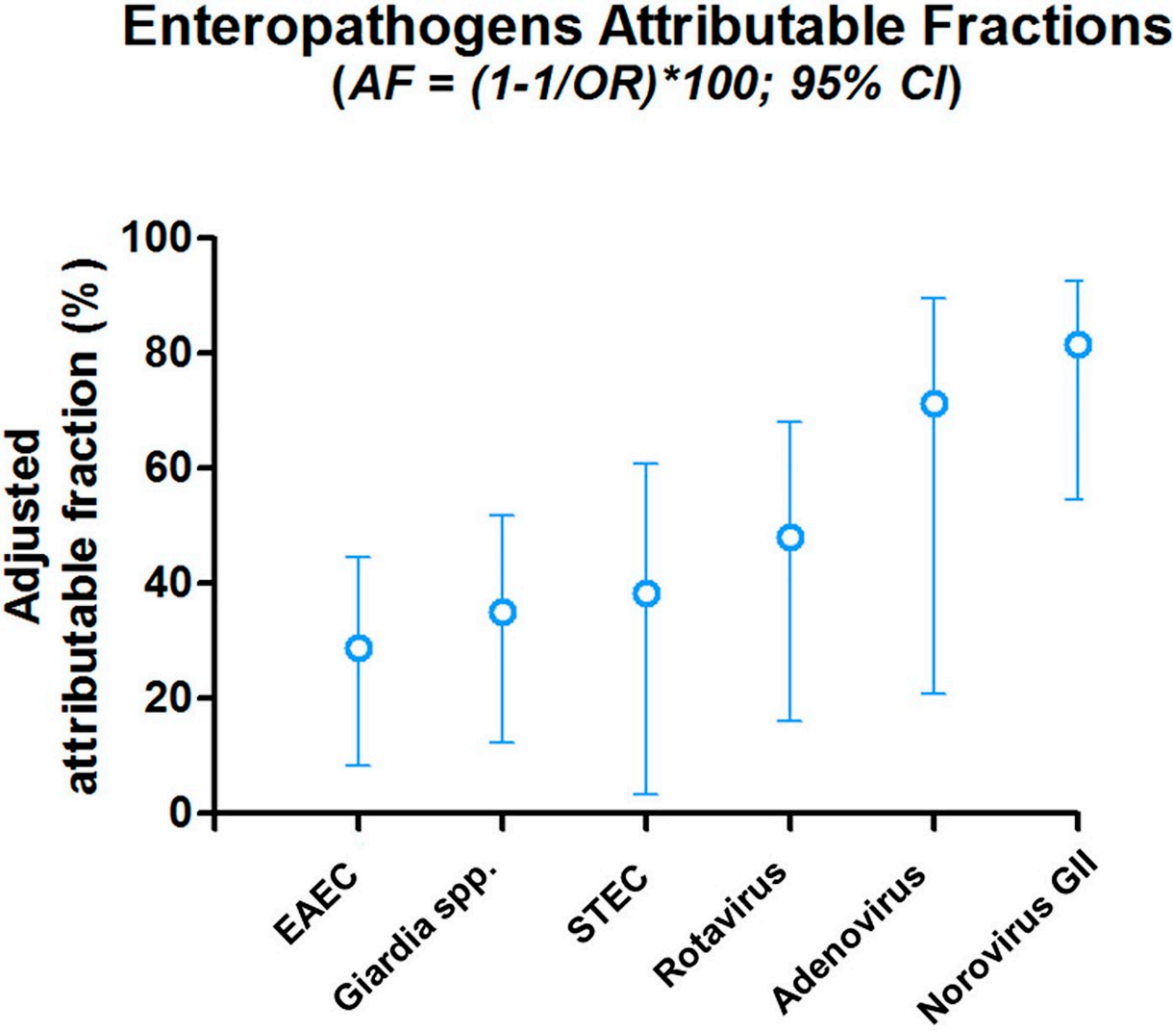
Multivariate logistic regression analysis showing the attributable fraction of enteropathogens association with diarrheal stool samples.

Since a higher proportion of these stool samples presented with two or more pathogens (55%; 661/1200), we adjusted the analysis using the multivariate logistic regression model to include all enteropathogens (Fig 3 and Table 3). The results show the most likely enteropathogens associated with diarrhea by decreasing odds ratio: norovirus GII (OR = 5.385; 95% CI: 2.196–13.203); adenovirus (OR = 3.476; 95% CI: 1.264–9.558); rotavirus (OR = 1.929; 95% CI: 1.192–3.121); shiga toxin-producing *E*. *coli* (STEC; OR = 1.623; 95% CI: 1.033–2.548); *Giardia* spp. (OR = 1.537; 95% CI: 1.140–2.074); and enteroaggregative *E*. *coli* (EAEC; OR = 1.403; 95% CI: 1.092–1.804). Fig 2D–2F) shows the pathogens prevalence by grouping children with one, two and three or more pathogens. In the group with one pathogen isolated, EAEC (50.7%; 180/355) had the highest prevalence (Fig 2D). EAEC (66.8%; 227/340), EPEC (38.2%; 130/340), and most atypical EPEC (35.6%; 121/340) had the highest prevalence when children presented with two pathogens, followed by *Giardia* spp. (25.1%; 85/338) (Fig 2E). Children with three or more pathogens had EAEC (75.7%; 243/321), EPEC (38.2; 13/340) and *Salmonella* spp. (36.6%; 117/420) as the most prevalent pathogens (Fig 2F).

**Table 3:** Multivariate logistic regression analysis of enteropathogens associated with diarrhea episodes.

Enteropathogens	Diarrhea episodes	
	OR (95% CI)	P values
Norovirus GII	5.385 (2.196–13.203)	0.000
Adenovirus	3.476 (1.264 9.558)	0.016
Rotavirus	1.929 (1.192–3.121)	0.007
STEC (*stx1* or *stx2)*	1.623 (1.033–2.548)	0.035
Giardia spp	1.537 (1.140–2.074)	0.005
EAEC (*aatA* or *aaiC)*	1.403 (1.092–1.804)	0.008
EPEC (*bfA* or *eaeA*)	1.017 (0.774–1.337)	0.904
ETEC (LT or ST; *eltB* or *estA)*	0.907 (0.602–1.365)	0.639
Astrovirus	1.400 (0.584–3.354)	0.450
Sapovirus	1.840 (0.877–3.858)	0.107
*Shigella* spp.	0.961 (0.638–1.449)	0.851
*Salmonella* spp.	0.858 (0.580–1.268)	0.442
*Campylobacter* spp.	1.481 (0.920–2.384)	0.106
*Aeromonas* spp.	1.198 (0.583–2.463)	0.623
*Entamoeba hystolitica*	1.059 (0.261–4.293)	0.936
*Cryptosporidium* spp.	1.171 (0.715–1.918)	0.531

All primers, polymerase chain reaction conditions and references are described in **S1 Table**.

To evaluate which of the enteropathogens were associated with higher severity, the type of episode and signs and symptoms of diarrheal episodes were analyzed using the multivariate logistic regression analysis model. Among all enteropathogens, enteroaggregative *E*. *coli* (EAEC) was the only one that maintained a significant association with severity of the diarrheal episodes (OR = 2.070; 95% CI 1.391–3.079). EAEC was also associated with moderate-to-severe dehydration (OR = 1.572; 95% CI 1.099–2.249). Norovirus GII was associated with fever (OR = 2.332; 95%CI 1.083–5.024). When we considered acute episodes (code = 0) and prolonged episodes (code = 1) in multiple regression analysis, norovirus GII was associated with prolonged diarrhea episodes (OR = 3.941; 95% CI 1.208–12.851) and *Salmonella* spp. was associated with acute episodes (OR = 0.044; 95% CI 0.012–0943).

## Discussion

This is the first large study on the broad etiology of diarrheal diseases using a highly sensitive and specific molecular diagnostic to identify causality and access both symptomatic and asymptomatic enteric infections in young children across six cities in the low-income semiarid region in Brazil. We were able to identify enteropathogens in 84.7% of the stool samples regardless of whether they were asymptomatic controls or symptomatic diarrhea cases. The study also showed that bacterial enteric infections were the most prevalent cause of diarrheal diseases, followed by protozoa and viruses. Overall, this report identified eight enteropathogens specifically associated with significant enteric infections in young children from this population: enteroaggregative *E*. *coli*, *Giardia* spp., shiga toxin-producing *E*. *coli*, rotavirus, norovirus GII, typical enteropathogenic *E*. *coli*, adenovirus, heat-stable and heat-labile producing enterotoxigenic *E*. *coli*.

Children in the first year of life had 56.4% of their diarrheal stool samples associated with two or more pathogens. We showed that symptomatic diarrheal cases had a significantly higher burden of two or more enteropathogens compared to asymptomatic controls. Kotloff et al., in a multisite matched case-control study (GEMS) in sub-Saharan Africa and south Asia, also found enteropathogen detection to be more common in diarrheal stools than non-diarrheal stools [10]. Similar results were found in the MAL-ED in South America, Africa, and Asia, where the number of enteropathogens detected was higher in diarrhea stools than non-diarrheal stools [21]. Multivariate logistic regression analysis of six enteropathogens: norovirus, adenovirus, rotavirus, STEC, *Giardia* and EAEC showed significant odds of being associated with a risk for diarrheal diseases. In the GEMS study they found rotavirus, *Cryptosporidium*, ETEC, tEPEC and *Shigella* as the major enteropathogens associated with moderate-to-severe diarrhea. Although we found similar enteropathogen association with diarrheal stools, such as rotavirus, ETEC and tEPEC, there are some differences due to different geographical areas, type of study design and selected child population.

The MAL-ED study identified norovirus GII, rotavirus, *Campylobacter* spp., astrovirus and *Cryptosporidium* spp. in the first year and *Campylobacter* spp., norovirus GII, rotavirus, astrovirus and *Shigella* spp. in the second year of life in these children. This report consistently showed a norovirus association with diarrheal stools in the first and second year of life in these children. The differences among other enteropathogens could be explained by different study designs and geographical areas.

EAEC was the most prevalent enteropathogen overall and by age at 2–11 months, 12–23 and 24–36 months, either alone or combined with other enteropathogens. In the MAL-ED study, EAEC also had a high prevalence reaching the second and third most prevalent enteropathogen in the first and second year of life, respectively (21). Recently, unpublished data, using a quantitative Real Time PCR approach in the surveillance of stools among 1,469 children from the MAL-ED cohort study identified EAEC as the most prevalent enteropathogen that also had an association with decrement in length at 2 years. In the same cohort study, Lima et al. also showed that EAEC subclinical infection and coinfection impaired child growth identified at the patient’s 6-month follow-up [22]. The children in the diarrhea case group of this report had a significantly lower length and head circumference compared to the control group children. Additional reports and recent studies showed that EAEC infections were associated with significant impact on child nutrition even in asymptomatic children, which is likely caused by gut inflammation and malnutrition [6,23,24]. This report also showed a consistently significant elevation of MPO, a marker of gut inflammation, in diarrhea stool samples compared to control group children. In the adjusted multivariate logistic analysis, EAEC also showed significant association with clinical severity of diarrhea cases, plus a specific association with moderate-to-severe dehydration. Lima et al. showed a combination of virulence genes, *aaiC* (aggR-activated island), and *agg3/4C* (usher, AAF/III-IV assembly unit), but lacking *agg4A* (AAF/IV fimbrial subunit), and *orf61* (cryptic protein) with diarrhea stools compared to control samples, which could contribute to understanding the pathobiology of EAEC enteric infection [25].

This study also showed that norovirus GII was associated with fever and prolonged episodes of diarrhea, while *Salmonella* spp. was associated with acute episodes. Rotavirus was also associated with diarrhea cases compared to controls, but this was in part due to lower vaccine coverage seen in this group even though the overall vaccine coverage rate was proportionally high. This brings attention to the value of increasing rotavirus vaccine coverage to prevent rotavirus enteric infection [26]. Norovirus GII and astrovirus enteric infections had a lower proportion of diarrhea cases, but they were associated with diarrheal stools in the univariate analysis. However, norovirus GII and adenovirus had the highest attributable fractions followed by rotavirus, STEC, *Giardia* spp. and EAEC. There are no vaccines for these enteric infections, except rotavirus, and the development of additional vaccines to prevent infection by norovirus GII, astrovirus and adenovirus would be an important contribution to global health [27,28]. *Giardia* spp. was more frequently associated with diarrhea cases when compared to controls in the adjusted analysis. *Giardia* spp. was the third most prevalent enteric infection after EPEC and EAEC. So, this is an important cause to be considered for enteric infections of both asymptomatic and symptomatic infections because of its negative impact on childhood malnutrition [29]. Consistent with the MAL-ED cohort study this report also showed ETEC (ST_LT producing) and STEC (shiga producing toxin *E*. *coli*) as important enteric infections significantly associated with diarrheal stools compared to control stools [21].

In the adjusted multivariate logistic analysis, we showed that only increased length of the child and number of rooms in the household were protective factors for diarrheal diseases. Lima et al. also reported an increased risk for EAEC infection and coinfections in non-diarrheal stools associated with lower socio-economic and sanitation facilities in the households of children (18). Baker et al. found sanitation and hygiene-specific risk factors for moderate-to-severe diarrhea in young children in the Global Enteric Multicenter Study [30].

The main limitation of this study was the case-control design, which by itself, limited the evaluation of diarrheal disease etiologies and asymptomatic carrier impact on nutrition and neurocognitive development among children as previously demonstrated decades ago and recently reported in MAL-ED cohort studies [13,14]. There are several advantages of this study, such as the molecular diagnostic approach utilized to study the etiologies of enteric infections, the sample size calculated to provide statistical significance to potentially lower prevalence enteric infections, and finally the identification of risk factors and common etiologies associated with enteric infections to facilitate planning for prevention and intervention via public health programs in low-income semiarid regions in Brazil.

In summary, these results show that bacterial diarrheal etiologies remain the most prevalent pathogens isolated from stool samples, followed by protozoa and viruses in the setting of young children from low-income semiarid regions in Brazil. In the adjusted multivariate logistic regression analysis, we identified six enteropathogens, norovirus, adenovirus, rotavirus, STEC, *Giardia* spp. and EAEC and odds associated with diarrhea cases compared to control children. These results suggest the importance of these six enteropathogens as causes of acute diarrhea episodes and of EAEC enteric infections having association with a high clinical severity score plus moderate-to-severe dehydration. Therefore, identifying key preventive measures and interventions that reduce exposure to these enteropathogens, developing and providing education for mother/caregiver, ensuring adequate child nutrition, increasing vaccination coverage, and improving access to better environmental and socio-economic factors are key to reduce diarrheal diseases and the potential consequences on growth and neurocognitive development for these children.

## Supporting information

S1 Checklist STROBE checklist. (DOCX)

S1 Table Organisms, target genes, primers, probes and PCR conditions. (DOCX)

S2 Table Multivariate logistic regression analysis of determinant variables associated with diarrhea episodes. (DOCX)

S3 Table Characteristics of the diarrhea episodes, severity, signals and symptoms association by cities from semiarid region in Brazil. (DOCX)

S4 Table Completed rotavirus and other vaccines covered in diarrhea cases and controls children at the time of enrollment in the study protocol. (DOCX)
