# Supplementary material for: Etiology and severity of diarrheal diseases in infants at the semiarid region of Brazil: A case-control study
Source: PLoS Negl Trop Dis. 2019 Feb 8;13(2):e0007154. doi: 10.1371/journal.pntd.0007154 (PMC6383952; doi:10.1371/journal.pntd.0007154)
Supplement: S1 Table — (DOCX) [file pntd.0007154.s002.docx]

**S1 Table.** Organisms, target genes, primers, probes and PCR conditions.

| **Organisms** | **Target gene**  **(Accession No.)** | **Primer/Probe Sequence ^1,2^** | | **PCR conditions**  **(40 cycles)** | **Reference** |
| --- | --- | --- | --- | --- | --- |
| ***Bacteria Panel*** |  |  |  |  |  |
| *EAEC^3^* | *aaiC-* *aggR-*activated island  (FN5547661) | F | ATTGTCCTCAGGCATTTCAC | 30 s at 94^o^ C | (1) |
|  |  | R | ACGACACCCCTGATAAACAA | 30 s at 60^o^ C | (1) |
|  |  | P | GTAGTGCATACTCATCATTTAAG | 60s at 72^o^ C | (1) |
| *EAEC* | *aatA-* anti-aggregation protein transporter  (AY351860) | F | CTGGCGAAAGACTGTATCAT | 30 s at 94^o^ C | (1) |
|  |  | R | TTTTGCTTCATAAGCCGATAGA | 30 s at 60^o^ C | (1) |
|  |  | P | TGGTTCTCATCTATTACAGACAGC | 60s at 72^o^ C | (1) |
| *EHEC^4^* | *stx1- Shiga*  toxin 1  (AE0051742) | F | ACTTCTCGACTGCAAAGACGTATG | 30 s at 94^o^ C | (1) |
|  |  | R | ACAAATTATCCCCTGAGCCACTATC | 30 s at 60^o^ C | (1) |
|  |  | P | CTCTGCAATAGGTACTCCA | 60s at 72^o^ C | (1) |
| *EHEC* | *stx2- Shiga*  toxin 2  (AE0051742) | F | GGCACTGTCTGAAACTGCTCC | 30 s at 94^o^ C | (1) |
|  |  | R | TCGCCAGTTATCTGACATTCTG | 30 s at 60^o^ C | (1) |
|  |  | P | GGGGAGAATATCCTTTAATA | 60s at 72^o^ C | (1) |
| *EHEC/EPEC^5^* | *eaeA-* Intimin adherence protein  (NC0116011) | F | GTAAAGTCCGTTACCCCAACCTG | 30 s at 94^o^ C | (1) |
|  |  | R | CAAAGCGCACAAGAYTACCA | 30 s at 60^o^ C | (1) |
|  |  | P | GCACATAAGCAGGCAAAATAGC | 60s at 72^o^ C | (1) |
| *EPEC* | *bfpA-* Bundle-forming pilus structural gene (Type 4 pilin)  (NC0116011) | F | GGAAGTCAAATTCATGGGGG | 30 s at 94^o^ C | (1) |
|  |  | R | GGAATCAGACGCAGACTGGT | 30 s at 60^o^ C | (1) |
|  |  | P | GCTGCAACCGTTACCGCAGG | 60s at 72^o^ C | (1) |
| *ETEC^6^* | *eltB –*Heat-labile enterotoxin B subunit  (NC0176331) | F | TTCCCACCGGATCACCAA | 30 s at 94^o^ C | (1) |
|  |  | R | CAACCTTGTGGTGCATGATGA | 30 s at 60^o^ C | (1) |
|  |  | P | CTTGGAGAGAAGAACCCT | 60s at 72^o^ C | (1) |
| *ETEC* | *estA –*Heat- stable enterotoxin  (NC0176331) | F | GCTAAACCAGTARGGTCTTCAAAA | 30 s at 94^o^ C | (1) |
|  |  | R | CCCGGTACARGCAGGATTACAACA | 30 s at 60^o^ C | (1) |
|  |  | P | TGGTCCTGAAAGCATGAA | 60s at 72^o^ C | (1) |
| *Shigella* spp./ EIEC*^7^* | *ipaH ^8^*- Invasion plasmid antigen  (M32063) | F | CGGAATCCGGAGGTATTGC | 30 s at 95^o^ C | (2) |
|  |  | R | CCTTTTCCGCGTTCCTTGA | 30 s at 60^o^ C | (2) |
|  |  | P | CGCCTTTCCGATACCGTCTCTGCA | 60s at 72^o^ C | (2) |
| *Aeromonas* spp. | *aerA-* plasmid-encoded  aerolysin  (M16495) | F | TYCGYTACCAGTGGGACAAG | 30 s at 95^o^ C | (2) |
|  |  | R | CCRGCAAACTGGCTCTCG | 30 s at 60^o^ C | (2) |
|  |  | P | CAGTTCCAGTCCCACCACTT | 60s at 72^o^ C | (2) |
| *Campylobacter jejuni* and *coli* | *cadF-* Campylobacter adhesion to fibronectin  (AF104303) | F | CTGCTAAACCATAGAAATAAAATTTCTCAC | 30 s at 95^o^ C | (2) |
|  |  | R | CTTTGAAGGTAATTTAGATATGGATAATCG | 30 s at 60^o^ C | (2) |
|  |  | P | CATTTTGACGATTTTTGGCTTGA | 60s at 72^o^ C | (2) |
| *Salmonella* spp. | *invA – Salmonella* invasion gene  (M90846) | F | TCGGGCAATTCGTTATTGG | 30 s at 95^o^ C | (2) |
|  |  | R | GATAAACTGGACCACGGTGACA | 30 s at 60^o^ C | (2) |
|  |  | P | GAAGACAACAAAACCCACCGCC | 60s at 72^o^ C | (2) |
| *Vibrio cholerae* | *toxR-*Cholera toxin transcriptional activator  (M21249) | F | GTTTGGCGWGAGCAAGGTTT | 30 s at 95^o^ C | (2) |
|  |  | R | TCTCTTCTTCAACCGTTTCCA | 30 s at 60^o^ C | (2) |
|  |  | P | CGCAGAGTMGAAATGGCTTGG | 60s at 72^o^ C | (2) |
| **Parasita Panel** |  |  |  |  |  |
| *Cryptosporidium* spp. | *COWP-* *Cryptosporidium* oocyst wall protein 1  (AF279916) | F | CAAATTGATACCGTTTGTCCTTCTG | 30 s at 95^o^ C | (3) |
|  |  | R | GGCATGTCGATTCTAATTCAGCT | 30 s at 55^o^ C | (3) |
|  |  | P | TGCCATACATTGTTGTCCTGACAAATTGAAT | 30s at 72^o^ C | (3) |
| *Entamoeba histolytica* | *18S rRNA-* 18S ribosomal RNA  (X641142) | F | AACAGTAATAGTTTCTTTGGTTAGTAAAA | 30 s at 95^o^ C | (3) |
|  |  | R | CTTAGAATGTCATTTCTCAATTCAT | 30 s at 55^o^ C | (3) |
|  |  | P | ATTAGTACAAAATGGCCAATTCATTCA | 30s at 72^o^ C | (3) |
| *Giardia lamblia* | *18S rRNA-* 18S ribosomal RNA  (M54878) | F | GACGGCTCAGGACAACGGTT | 30 s at 95^o^ C | (3) |
|  |  | R | TTGCCAGCGGTGTCCG | 30 s at 55^o^ C | (3) |
|  |  | P | CCCGCGGCGGTCCCTGCTAG | 30s at 72^o^ C | (3) |
| Extrinsic control (Phocine Herpes Virus) | *gB^9^* -Glycoprotein B 16  (Z68147) | F | GCGGTTCCAAACGTACCAA | 30 s at 94^o^ C  30 s at 95^o^ C | (1,2,3) |
|  |  | R | GGGCGAATCACAGATTGAATC | 30 s at 60^o^ C  30 s at 55^o^ C | (1,2,3) |
|  |  | P | TATGTGTCCGCCACCATCTG | 60s at 72^o^ C  30s at 72^o^ C | (1,2,3) |
| ***Virus Panel*** |  |  |  |  |  |
| Adenovirus | *Hexon- capsid protein*  (L19443 and M21163) | F | GCCACRGTGGGRTTTCTCAACTT | 30 s at 94^o^ C | (4) |
|  |  | R | GCCGCAATGGTCTTACATGCACATC | 30 s at 60^o^ C | (4) |
|  |  | P | TGCACCAGGCCCGGGCTCAG | 60s at 72^o^ C | (4) |
| Astrovirus | *Capsid*  (AY720892) | F | CAGTTGCTTGCTGCGTTCA | 30 s at 94^o^ C | (4) |
|  |  | R | CTTGCTAGCCATCACACTTCT | 30 s at 60^o^ C | (4) |
|  |  | P | CACAGAAGAGCAACTCCATCGC | 60s at 72^o^ C | (4) |
| Norovirus GII | *ORF1–ORF2 – Open reading frame1 and 2*  (AF145896) | F | CARGARBCNATGTTYAGR | 30 s at 94^o^ C | (4) |
|  |  | R | TGGATGAGTCGACGCCATCTTCATTCACA | 30 s at 60^o^ C | (4) |
|  |  | P | TGGGAGGGCGATCGCAATCT | 60s at 72^o^ C | (4) |
| Rotavirus | *NSP3- non-structural protein 3*  (X81436) | F | ACCATCTWCACRTRACCCTCTATGAG | 30 s at 94^o^ C | (4) |
|  |  | R | GGTCACATAACGCCCCTATAGC | 30 s at 60^o^ C | (4) |
|  |  | P | AGTTAAAAGCTAACACTGTCAAA | 60s at 72^o^ C | (4) |
| Saprovirus | *RdRp-capsid^10^*  (AY237420, U73124 and  AY646856 ) | F1 | GAYCAGGCTCTCGCYACCTAC | 30 s at 94^o^ C | (4) |
|  |  | F2 | TTGGCCCTCGCCACCTAC | 30 s at 60^o^ C | (4) |
|  |  | R1 | TTTGAACAAGCTGTGGCATGCTAC | 60s at 72^o^ C | (4) |
|  |  | R2 | CCCTCCATYTCAAACACTA |  | (4) |
|  |  | P1 | CYTGGTTCATAGGTGGTRCAG |  | (4) |
|  |  | P2 | CAGCTGGTACATTGGTGGCAC |  |  |
| Extrinsic control | *MS2g1-* Bacteriophage MS2  (AY237420) | F | TGGCACTACCCCTCTCCGTATTCAC | 30 s at 94^o^ C | (4) |
|  |  | R | GTACGGGCGACCCCACGATGAC | 30 s at 60^o^ C | (4) |
|  |  | P | CACATCGATAGATCAAGGTGCC | 60s at 72^o^ C | (4) |

^1^ Mixed bases are as follows: Y, C or T; R, A or G; B, not A; N, any.; ^2^ F, R and P represent forward primer, reverse primer-- biotinylated, and capture oligonucleotide probe, respectively.; ^3^ *EAEC*- enteroaggregative *Escherichia coli;* ^4^ *EHEC*- enterohemorrhagic *Escherichia coli;* ^5^ *EPEC*- enteropathogenic *Escherichia coli;* ^6^ *ETEC*- enterotoxigenic *Escherichia coli;* ^7^ *EIEC*- enteroinvasive *Escherichia coli;* ^8^ The *ipaH* gene was targeted for detection of both *Shigella* and EIEC; *^9^ gb-Glycoprotein B 16* was used as extrinsic control to panels of bacteria and parasite; ^10^*RdRp-* RNA-dependent RNA polymerase-capsid.
