# Supplementary material for: Etiology and severity of diarrheal diseases in infants at the semiarid region of Brazil: A case-control study
Source: PLoS Negl Trop Dis. 2019 Feb 8;13(2):e0007154. doi: 10.1371/journal.pntd.0007154 (PMC6383952; doi:10.1371/journal.pntd.0007154)
Supplement: S2 Table — (DOCX) [file pntd.0007154.s003.docx]

**S2 Table.** Multivariate logistic regression analysis of determinant variables associated with diarrhea episodes.

| **Variables** | **Diarrhea episodes** | | | |
| --- | --- | --- | --- | --- |
|  | **β Coefficients** | **Standard errors** | **Odd ratio (95% CI)** | **P values** |
| Age (months; mean ± sem) | 0.011 | 0.014 | 1.011 (0.984 – 1.038) | 0.447 |
| Current weight of the child (mean ± sem) | 0.029 | 0.043 | 1.030 (0.947 – 1.121) | 0.494 |
| Current length of the child (mean ± sem) | -0.040 | 0.016 | 0.960 (0.931 – 0.991) | 0.011 |
| Current head circumference (mean ± sem) | -0.026 | 0.031 | 0.974 (0.917 – 1.034) | 0.386 |
| Age of the mother at child enrollment | -0.013 | 0.012 | 0.987 (0.964 – 1.012) | 0.310 |
| Age of your first pregnancy? (mean ± sem) | -0.006 | 0.016 | 0.994 (0.962 1.026) | 0.694 |
| How many rooms are there in your household? (mean ± sem) | -0.113 | 0.041 | 0.893 (0.824 – 0.968) | 0.006 |
| How many people usually sleep in this household? (mean ± sem) | 0.001 | 0.040 | 1.001 (0.925 – 1.082) | 0.988 |
| How many children less than 5 years old sleep in this household? (mean ± sem) | 0.134 | 0.118 | 1.144 (0.907 – 1.442) | 0.256 |
| What is the average monthly income for the entire household? (mean ± sem) | 0.040 | 0.060 | 1.041 (0.925 – 1.171) | 0.503 |
