# Supplementary material for: Etiology and severity of diarrheal diseases in infants at the semiarid region of Brazil: A case-control study
Source: PLoS Negl Trop Dis. 2019 Feb 8;13(2):e0007154. doi: 10.1371/journal.pntd.0007154 (PMC6383952; doi:10.1371/journal.pntd.0007154)
Supplement: S3 Table — (DOCX) [file pntd.0007154.s004.docx]

**S3 Table.** Characteristics of the diarrhea episodes, severity, signals and symptoms association by cities from semiarid region in Brazil.

| **Cities, States at semiarid region in Brazil** | **Diarrhea episodes**  **N** | **Acute**  **N (%)** | **Prolonged**  **N (%)** | **Diarrhea severity^*^**  **N (%)** | **Abdominal**  **pain**  **N (%)** | **Blood in stools**  **N (%)** | **Associated fever**  **N (%)** | **Associated vomiting**  **N (%)** | **Associated dehydration**  **N (%)** |
| --- | --- | --- | --- | --- | --- | --- | --- | --- | --- |
| **Crato, Ceará** | 100 | 100/100  (100) | 0/100  (0) | 11/100  (11) | 7/100  (7) | 0/100  (0) | 25/99  (25) | 16/100  (16) | 15/100  (15) |
| **Picos, Piauí** | 97 | 90/92  (98) | 2/92  (2) | 12/95  (13) | 43/95  (45) | 4/95  (4) | 12/95  (13) | 32/95  (34) | 55/93  (57) |
| **Ouricuri, Pernambuco** | 100 | 99/100  (99) | 1/99  (1) | 11/100  (11) | 23/100  (23) | 1/99  (1) | 20/98  (20) | 25/100  (25) | 14/99  (14) |
| **Cajazeiras, Paraíba** | 100 | 83/99 (84%) | 16/99  (16) | 43/100  (43) | 12/100  (12) | 0/100  (0) | 61/100  (61) | 33/100  (33) | 43/100  (43) |
| **Patos, Paraíba** | 100 | 98/100  (98) | 2/100  (2) | 53/100  (53) | 1/100  (1) | 0/100  (0) | 38/100  (38) | 40/100  (40) | 60/100  (60) |
| **Sousa, Paraíba** | 99 | 89/99  (90) | 10/99  (10) | 50/99  (51) | 10/99  (10) | 0/99  (0) | 45/99  (46) | 43/99  (43) | 55/99  (56) |
| **Total** | 596 | 559/590  (95) | 31/590  (5) | 180/594  (30) | 96/594  (16) | 5/593  (1) | 201/591  (34) | 189/594  (32) | 242/591  (41) |

**^*^** Elements derived from the MAL-ED score (2).
