# Supplementary material for: Etiology and severity of diarrheal diseases in infants at the semiarid region of Brazil: A case-control study
Source: PLoS Negl Trop Dis. 2019 Feb 8;13(2):e0007154. doi: 10.1371/journal.pntd.0007154 (PMC6383952; doi:10.1371/journal.pntd.0007154)
Supplement: S4 Table — (DOCX) [file pntd.0007154.s005.docx]

**S4 Table.** Completed rotavirus and other vaccines covered in diarrhea cases and controls children at the time of enrollment in the study protocol.

| **Type of vaccines** | **Total**  **N = 1200** | **Diarrhea**  **N = 596** | **Controls**  **N = 604** | ***P* values** | **OR** | **95% CI** |
| --- | --- | --- | --- | --- | --- | --- |
| **Rotavirus (Rotarix^®^ G1P[8] GlaxoSmithKline, Wavre, Belgium)** | 1113 (93) | 537 (90) | 576 (95) | 0.001 | 0.715 | 0.610 – 0.839 |
| **BCG, Bacillus Calmette-Guérin** | 1196 (100) | 594 (100) | 602 (100) | 1.00 | 1.010 | 0.063 – 16.187 |
| **MMR, Measles, Mumps and Rubeola** | 811 (68) | 375 (63) | 436 (72) | 0.001 | 0.815 | 0.727 – 0.914 |
| **Hepatite B** | 1041 (87) | 483 (81) | 558 (92) | <0.001 | 0.655 | 0.581 – 0.738 |
| **Hib, *Haemophilus influenzae type b*** | 1027 (86) | 477 (80) | 550 (91) | <0.001 | 0.677 | 0.600 – 0.764 |
| **DPT, Diphtheria, Tetanus and Pertussis** | 689 (58) | 309 (52) | 380 (63) | <0.001 | 0.800 | 0.714 – 0.895 |
| **OPV, Oral Polio Vaccine** | 689 (58) | 310 (52) | 379 (63) | <0.001 | 0.805 | 0.719 – 0.901 |

Rotarix (2 and 4 months); BCG (after birth); MMR (12 months); Hepatite B (1 and 4 months); Hib (2, 4 and 6 months); DPT (2, 4, 6 and 15 months); OPV (2, 4, 6 and 15 months).
